# Wounds of the Heart

**Published:** 1828-10

**Authors:** 


Art. II.—*Wounds of the Heart.* Translated from the Dictionaire de Sciences Medicales, vol. 43.

The foregoing case, so far as it relates to the wound of the heart at least, is not without interest. Supposing that some of our readers might be disposed to inquire, whether wounds of that organ have generally proved fatal, and whether the records of surgery furnish a parallel case to that reported by Dr. Randal, we have been induced to refer to that learned, circumstantial, and comprehensive work, the great French Dictionary of the Medical Sciences, and translate for this Journal, the article, *Wounds of the Heart.* It will be seen that it mentions no case similar to the above; nor do we recollect to have met with the report of any case in which an *extraneous* body resided, even for a short time, in the cavities of the heart. Dr. Randal's patient seems to have died from the wound of the lungs rather than that of the heart; though had the former been uninjured, he doubtless would have died, but at a more distant time, from disease of the latter. His death, however, would have been the immediate consequence, not of the wounds of the ventricle, but of the inflammation of its internal surface, occasioned by the mechanical action of the shot lodged within it. Had they passed out, and the lungs remained unhurt, the patient would probably have survived the accident. The size of the shot is not mentioned, but it may be presumed that they were small.—Ed.

### Translation.

*Wounds of the Heart.* In wounds penetrating the cavity of the chest, the heart may be injured, and its læsion is generally mortal; it is not, however, necessarily fatal if the foreign body has not penetrated into its cavities, as we may be convinced by reading the Treatise of Senac upon the structure and diseases of the heart, 2 volumes in 4to. A posthumous case of Wolff seems to prove, that there had been a cure in a man who had formerly received a wound in the heart. If we may credit the reports contained in the *Melanges des curieux de la nature*, cicatrices have been seen upon the hearts of domestic animals; and some observers declare that they have even met with them in man. M. Richerand, *Nosogr. chirurg. tom. iv*, relates that in dissecting the body of an individual who had once received the thrust of a sword above the left hypochondrium, he found the pericardium adhering to the heart by a cicatrix, which was itself attached to the parieties of the left ventricle.

Whenever an instrument wounds the base of the heart, death inevitably follows; for it is from this part that the great bloodvessels issue. In every case of this kind, some one of them must be injured, and with the blood that issues, life itself escapes: we may add that it is at the base of this organ, that the great cardiac plexus of nerves is spread out.

Numerous observations prove that men have lived for many days after receiving very deep wounds of the heart; some penetrating into one of the ventricles, others piercing both ventricles, and traversing the whole organ. Saviard cites the case of a man who had his heart pierced through and through, and who still lived from four to five days. On opening the body after death, Saviard found, that both the left ventricle and the *septum* which separates it from the right, had been perforated; and that some *coagula* of blood had stopped up the wound. A young man, according to Thomas Bartholin, was wounded between the third and fourth ribs of the left side; he was able to return to his house, at a considerable distance from the place where he was stabbed, and survived, for five days, a narrow wound of the right ventricle.

In 1735, Morand exhibited to the Academy of Sciences, the heart of a soldier, who died at the Hospital of Charity, of a sword wound in the anterior and lateral part of the left side of the chest. For three days he did not experience any violent symptoms: a fever supervened on the fourth, with difficulty of respiration, and he died on the ninth. The sword had passed through the pericardium, the inferior part of the right ventricle, the diaphragm, and the liver. We read in the *Nouvelle Doctrine Chirurgicale*, of M. Leveille, the history of a young man, the left ventricle of whose heart, pierced vertically, received a wound three or four lines in length: he expired the seventh day. Dehors, Rhodius, and Fantoni, have seen individuals, wounded in the heart, survive fourteen, sixteen, seventeen, and even twenty days. To explain this phenomenon, some have conjectured that in the cases just cited, the wounds were so small that the blood only escaped *guttatim*, and that there formed in them a clot, the detachment of which at the end of one or many days, was followed by death. Others have supposed that the wounds were superficial, and that there remained a layer of fibres sufficiently strong to resist for a time, the escape of the blood; but that they were at length ruptured by the contraction of the heart, and the hæmorrhage which ensued, occasioned immediate death. Saucarette relates, in the *Melanges de Chirurgie*, a case reported by Levoyer, in which it was established, that the tricuspid valves, had given birth to a fatty body, which, by its interposition between the lips of a wound of the right ventricle, prevented the hæmorrhage, in a soldier, who lived for two days after the accident.

Ambrose Parc has recorded, that a gentleman, at Turin, when engaged in single combat, received a thrust of a sword under the left breast, upon which he discontinued the contest, but walked two hundred steps and then expired. The heart presented a wound, which would receive the end of the finger, and a great deal of blood was extravasated upon the diaphragm. The two ventricles were found pierced through and through in a student of Ingolstadt, who, says Schenk, having received the puncture of a stylet, in the left side of the chest, walked a considerable distance, preserved his presence of mind during an hour, recommended himself to God, conversed and then died. Is it not probable in this case, that the instant the wound was inflicted, the heart contracted spasmodically, and thus closed the wound, and prevented the immediate escape of the blood? The hæmorrhage would take place, when the spasm having ceased, the wound would become extended to its proper dimensions.

M. Sue (*Recueil periodique de la Societe de Medicine de Paris,* viij p. 31) reports the history of a woman, who pierced the heart of her husband, when asleep, with a very long and sharp golden pin, which she had made expressly for the purpose. The right ventricle was penetrated completely through. She acknowledged her attrocious crime and was condemned to death.

The diagnostic of wounds of the heart is very difficult. In fact, their situations, a knowledge of the depth to which the instrument penetrated the chest, the abundant flow of blood, the trembling of the whole body, the faintness, the smallness and inequality of the pulse, the cold sweats, the anxiety, the coldness of the limbs, the difficulty of respiration, sometimes palpitations, and fever if life be prolonged, are symptoms common to wounds of the great vessels, of the pericardium, and of the diaphragm. When many days elapse between the infliction of the wound and the death of the patient, it is difficult to predict the state in which the heart will be found.

A wound of one of the coronary arteries may be mistaken for a wound of the heart. In the month of August 1697, Lamotte saw the captain of a regiment, Lamar, who received, behind, a thrust of a sword, between the fifth and sixth left ribs, the point of the instrument made its exit, a little above the nipple of the same side. The captain, cold and without pulse, expired two hours afterwards. They found the pericardium opened in two places; and an oblique wound, not penetrating the heart, extended through the coronary artery: The cavity of the thorax was full of blood.

When wounds of the heart have a certain extent, they produce immediate death; if they are narrow, the life of the patient may be prolonged for some days.

What treatment can be opposed to such a wound? If it should not be speedily mortal, we ought to diminish the quantity of blood by copious venesection, administer antispasmodics, and keep the patient in the most perfect repose. We should be careful, not to probe or sound the wound, for fear of detaching the clot, and thus removing the only obstacle which opposes some resistance to the flow of the blood.

